# A dataset on human navigation strategies in foreign networked systems

**DOI:** 10.1038/sdata.2018.37

**Published:** 2018-03-13

**Authors:** Attila Kőrösi, Attila Csoma, Gábor Rétvári, Zalán Heszberger, József Bíró, János Tapolcai, István Pelle, Dávid Klajbár, Márton Novák, Valentina Halasi, András Gulyás

**Affiliations:** 1 MTA-BME Information Systems Research Group, Department of Telecommunications and Media Informatics, Budapest University of Technology and Economics, H-1117 Budapest, Magyar tudósok krt. 2, Hungary; 2 Eötvös Loránd University, Department of Operations Research, H-1117 Budapest, Pázmány Péter sétány 1/C, Hungary

**Keywords:** Human behaviour, Language, Problem solving

## Abstract

Humans are involved in various real-life networked systems. The most obvious examples are social and collaboration networks but the language and the related mental lexicon they use, or the physical map of their territory can also be interpreted as networks. How do they find paths between endpoints in these networks? How do they obtain information about a foreign networked world they find themselves in, how they build mental model for it and how well they succeed in using it? Large, open datasets allowing the exploration of such questions are hard to find. Here we report a dataset collected by a smartphone application, in which players navigate between fixed length source and destination English words step-by-step by changing only one letter at a time. The paths reflect how the players master their navigation skills in such a foreign networked world. The dataset can be used in the study of human mental models for the world around us, or in a broader scope to investigate the navigation strategies in complex networked systems.

## Background & Summary

The way people navigate among the world's physical objects is of crucial importance from the perspective of their survival. Due to their crucial function, the navigation strategies of humans have been extensively studied in the past^1–7^. Some more recent studies have identified similarities between physical and social navigation strategies and hint that people treat the “Where am I?” (i.e. How do I identify myself in the network of physical objects?) question very similarly to the “Who am I?” (i.e. How do I locate myself in the network of social contacts?) question from a navigational point of view^8–11^. In our modern world it is not necessary to stress that the proper usage of the social network is at least as important as navigating in the physical world from the perspective of survival. Very recently Csoma et. al. have put navigation into an even broader prospect. They have found similar navigation strategies in networks coming from very diverse areas of life such as the human brain, the Internet, airport networks and word networks^12^. In particular, Csoma et. al. analyzed how people learn to navigate in a special network of English words, the word morph network where fixed-length English words are connected if they differ only in a single letter. They found that people abstract a hierarchy (similarly to social navigation) out of this network and use that as a mental model when solving puzzles on the network. The database leading to these findings is presented and shared in this paper. We believe that further studying of the navigation strategies of people in any kind of networked systems (physical world, social contacts, language) will uncover unexpected similarities and lead to a better understanding of how our brain learns and navigates in a foreign networked world.

**Figure 1:**
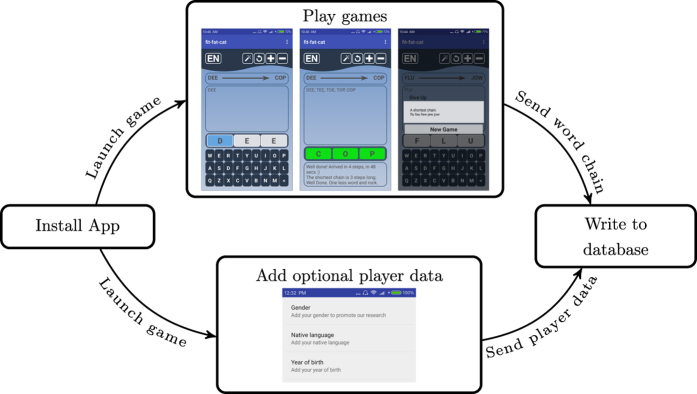
Data gathering process

Our database has been collected by a smartphone application called “fit-fat-cat”^13^. After the users install the game (see Figure 1), they are asked to transform a three-letter English source word into a three-letter target word through meaningful intermediate three-letter English words by changing only a single letter at a time. The word chain fit-fat-cat is a good solution of a game with source word ‘fit’ and target word “cat”. These word chains are collected anonymously, with some other optional metadata about the players (gender, language, year of birth). The word chains in the database can be considered as the footprints of human navigation over the word morph network of the English language and may be good sources for studying human navigation strategies in the future.

Here we identify two possible future directions where the shared data could be used. (i) The word morph network is almost surely unknown for most people before playing the game. One may reason that native English speakers have significant advantages but it seems from the data that this is not the case. In fact the learning curve for native and non-native speakers seem to be very similar. Since the game records are timestamped, the dataset may be a good candidate source for analyzing how people find their way in a foreign networked system, how fast they build up a mental model for a basically unknown networked world, what characteristic features such models may have and how that model changes in time. (ii) Secondly, the in-depth analysis of the paths used by people after mastering the game can give valuable information about other systems navigated by humans day-by-day. Cognitive psychology, neuroscience, public transportation services, social and organizational networks are all imaginable areas that could possibly benefit from such information.

## Methods

The dataset reported in this paper is collected via a smartphone application called “fit-fat-cat” running on most Android phones and tablets. The application is available from the Google Play store^13^ and the installation is as easy as any other apps. After installing and launching the application, the word morph game is ready to play. By playing the game, the players give their consent to openly use their anonymized data without restrictions.

The screenshots in the “Play games” box of Figure 1 show the main screen of the application. When a player starts a game the source and target words are randomly picked from the all possible three-letter English words. The source and target words are displayed in a box with an arrow pointing from the source (DEE in the example of Figure 1) to the target word (COP). Below this box one can see one's chain entered so far. When starting a new game the chain contains only the source word (DEE). The player can enter the consecutive words in a user friendly manner by using a virtual keyboard of the phone. First they select which letter they want to change (D in the example) than they choose the new letter with the keyboard (see the left screenshot in the “Play games” box of Figure 1). After changing a letter, the app automatically adds the new word to the chain. This way the players can see which words they have already tackled when solving a word puzzle. The game may end in three ways. If the player can reach the target word through such one-letter transformations the puzzle is solved. In this case, the word becomes green-colored to show the end of the game and the player gets some motivating messages at the bottom of the screen (see the middle screenshot in the “Play games” box of Figure 1). Secondly, the player can give up the game by pressing the “new game” button represented by a circle arrow to the left of the + and − buttons at the top of the screen. In this case the player gets the next puzzle automatically. Finally, the player can press the “magic wand” button to the left of the “new game”. In this case a possible (optimal, i.e. shortest path) solution of the puzzle is shown before starting a new game (see the right screenshot in the “Play games” box of Figure 1). No matter how the game is ended, the chain field (along with some other metadata, see the Data Records section) is anonymously submitted to our database stored in the cloud. Thus the application collects also the chains which are not completed, i.e. their last word is not the target word.

The application is capable of generating puzzles with longer words and also in other languages. The word length can be increased and decreased by pressing the + and − buttons respectively in the top left corner of the screen. The language indicator in the top right corner (displaying EN in the screenshots of Figure 1) shows the current language, which can be changed by pressing the button. These buttons are activated only after playing fifty successful games with the three-letter English version. Note that this manuscript describes the three-letter data, but the games with longer words are also shared in the dataset.

For providing a better gaming experience to the players, they can obtain badges for their achievements and can write themselves up to leaderboards. Badges are given for playing certain number of games, for optimal solutions or for very fast solutions. The achievements and leaderboards can be accessed by pressing the “Menu” button represented by ⋮ (vertical dots) in the top left corner of the application.

In addition to collecting the chains, the application also collects optional data about the players. Gender, language and year of birth can be given in a form (see the screenshot in the “Add optional player data” box of Figure 1) and players willing to disclose these pieces of information earn the “Open person” badge. The application is collecting data since October 2016. The collected dataset is available at Data Citation 1.

### Interpretation of the data

**Figure 2:**
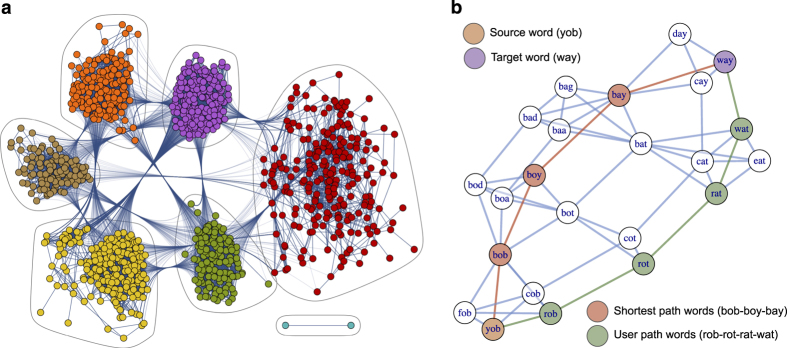
The word morph network. Panel (**a**) shows the whole word morph network in a clustered layout. The word morph network is a network of three-letter English words, in which two words are connected by a link if they differ only in a single letter. For example ``FIT'' is linked to ``FAT'' as they differ only in the middle letter, but ``FIT'' and ``CAT'' are not neighbors in this network since more than one letters differs in them. The network has a giant connected component and a small cluster containing only two nodes. Panel (**b**) shows a word morph game example with source and target words ``YOB'' and ``WAY''. A shortest path solution is displayed in red, while a solution given by a specific player is shown in green. For the sake of clearer context, the plot shows a larger subgraph of the word morph network with nodes and links colored in white and blue respectively.

The collected chains are footprints of the process how people master their navigational skills in the network lying behind the game. The word morph network is a network of three-letter English words, in which two words are connected by a link if they differ in only a single letter at the same position (see Figure 2a). For example the word “FIT” is connected to the word “FAT” as they differ only in their middle letter. “FAT” is linked to “CAT” as they differ in their first letter, but “FIT” and “CAT” are not connected in this network since they differ in more than one letter. The chains collected from players are paths in this network and reflect valuable information about how people try to navigate between nodes. Figure 2b shows a subgraph of the word morph network and illustrates two solutions for a game between source and target words “YOB” and “WAY”.

### Code availability

The fit-fat-cat application collecting the data is available at the Google Play store^13^. The source code of the app can be requested via e-mail to the corresponding author.

## Data Records

Our dataset (available at Data Citation 1) is organized into three files. The file named “three_letter_scrabble_words.txt” contains the 3-letter long official word list for Scrabble as of 2016. From this list one can reconstruct the word morph network (see Figure 2) containing the 3-letter English words as nodes that are connected if they differ in exactly one letter. The graph representation of this network is provided under the file “word_morph_network.gml” in the GML format^14^.

The third file named “word_navigation_game_export.json” is the dataset containing information about the word games as played by our players along with some additional meta data about the players themselves the provision of which is optional. All these data is organized into one JSON^15^ formatted file containing 19828 game records gathered from our players. Game records are stored under the object name “GameLogs” and grouped by players using anonymous player identifiers (28 character long hash strings). Each game record is identified by a 20 letter long hash code and contains eight fields as detailed in Table 1. The player records are stored under the object name “Users”, each player is identified by a 28 letter long hash string. Gender, language and date of birth are collected from players who are willing to share them (not mandatory for playing the game). The corresponding fields in the dataset are described in Table 2.

## Technical Validation

The collection of the game data is fully automated by the “fit-fat-cat” application. After the players finish the game, the application automatically uploads the game record to our server. Only the “chain” field is collected directly from the player, all other information (date, time taken to complete the game, source word, target word, chain length etc.) are filled out automatically by the application. The collection process of the optional data about the players uses standard techniques to increase the quality of the data (e.g. gender and language information can be chosen from lists).

The application chooses source and target words in a randomized fashion, which ensures that the data is not biased towards specific words and contains navigation paths (i.e. game solutions) covering all regions of the word morph network. The dataset is fully anonymized, players and games are identified by hash codes, which shades the identity of the players but eases the processing of the data.

## Usage Notes

The shared dataset is easy to read and process by basic software tools. The file “three_letter_scrabble_words.txt” holding the list of 3-letter long official words for Scrabble can be read by an arbitrary text editor. The word morph network provided under the file “word_morph_network.gml” can be parsed by ordinary GML parsers (e.g. by using igraph's^16^ GML interface either from Python or R codes). The game data shared under the file named “word_navigation_game_export.json” can be parsed by standard JSON^15^ parsers. Exemplary code snippets are displayed in Supplementary Table 1 to show how to parse and use the database from Python or R codes. Some basic statistics (number of records and the mean value of the word chain lengths entered by the players) are also computed to illustrate the usage of the data.

## Additional information

**How to cite this article:** Kőrösi, A. *et al.* A dataset on human navigation strategies in foreign networked systems. *Sci. Data* 5:180037 doi: 10.1038/sdata.2018.37 (2018).

**Publisher’s note:** Springer Nature remains neutral with regard to jurisdictional claims in published maps and institutional affiliations.

## Supplementary Material



Supplementary Information

## Figures and Tables

**Table 1:** Data records for the games played.

*chain*	(string) Ordered sequence of 3-letter words (strings separated by spaces) given by the player to solve a game. The first word is always the one that is given as the source word, later ones are chosen by the player. If the last word is not the target word the game is unfinished, most likely the player gave it up.
*chain_length*	(integer) The number of 3-letter words in the *chain* field. It is added to ease the use of the dataset.
*date*	(string) The date of the game played.
*language*	(string) The language of the current game.
*sourceWord*	(string) The starting 3-letter word dispatched randomly by the application for the current game. It is always the first word in the *chain* field.
*targetWord*	(string) The target 3-letter word dispatched randomly by the application for the current game. If it corresponds to the last word in the *chain* field than the game is completed successfully.
*time_in_sec*	(integer) The duration of the game as played by the player in seconds. If the game was successful it indicates the total time taken by the player to find path between the source and target word. If the game was unfinished it indicates the time when it was given up.
*wordlength*	(integer) Indicates the length of the words (3 for the three-letter games).

**Table 2:** Data records for the players.

*gender*	The gender of the player (string: 'Male' or 'Female')
*language*	Nationality of the player (string) e.g. 'English'
*yearofbirth*	Year of birth of the player (four digit integer)
