# Supplementary material for: A dataset on human navigation strategies in foreign networked systems
Source: Sci Data. 2018 Mar 13;5:180037. doi: 10.1038/sdata.2018.37 (PMC5848790; doi:10.1038/sdata.2018.37)
Supplement: Supplementary Information [file sdata201837-s2.pdf]

Supplementary information for: A dataset on human navigation strategies in foreign networked systems

Attila Kőrösi, Attila Csoma, Gábor Rétvári, Zalán Heszberger,
József Bíró, János Tapolcai, István Pelle, Dávid Klajbár,
Márton Novák, Valentina Halasi and András Gulyás
Corresponding author: András Gulyás (gulyas@tmit.bme.hu)

Supplementary Tables

Python	R

Read and parse database

Python

```
import json
import numpy

dataSet = "word_navigation_game_export.json"

def importDataset(dataSet):
    data = json.load(open(dataSet))
    return data

dbase = importDataset(dataSet)
print(json.dumps(
  dbase["GameLogs"].values()[10].values()[5]))
```

```
Output:
{"chain": "RAN RAS PAS PIS XIS", "language": "EN",
"targetWord": "XIS", "time_in_sec": 32,
"wordlength": 3, "date":
"Thu Nov 17 07:57:38 GMT+01:00 2016",
"chain_length": 5, "sourceWord": "RAN"}
```

R

```
library("rjson")

dataSet <- "word_navigation_game_export.json"

importDataset <- function(dataSet){
    parsedData <- fromJSON(file = dataSet)
}

dbase <- importDataset(dataSet)

## print the second player's first game record
print(dbase$GameLogs[[2]][[1]])
```

```
Output:
$chain
[1] "ADD AID AIR AIT AFT"
$chain_length
[1] 5
$date
[1] "Thu Nov 10 22:50:50 CET 2016"
$language
[1] "EN"
$sourceWord
[1] "ADD"
$targetWord
[1] "AFT"
$time_in_sec
[1] 51
$wordlength
[1] 3
```

Basic statistics

Python

```
records = 0
chLengths = []
for playerData in dbase["GameLogs"].values():
    for game in playerData.values():
        records += 1
        chLengths.append(game["chain_length"])

print("Number␣of␣records", records)
print("Mean␣chain␣lenght",
                       numpy.mean(chLengths))
```

```
Output:
('Number of records', 19828)
('Mean chain lenght', 3.8835485172483355)
```

R

```
records <- 0
chLengths <- c()
for (playerData in dbase$GameLogs){
    for (game in playerData){
        records <- records + 1
        chLengths <- append(chLengths,
                            game$chain_length)
    }
}

cat("Number␣of␣records␣",records,"\n")
cat("Mean␣chain␣lenght␣",mean(chLengths),"\n")
```

```
Output:
Number of records  19828
Mean chain lenght  3.883549
```

Table 1: Usage of the game database from Python and R.
